# Discordant intestinal malrotation in adult monozygotic twins discovered incidentally during laparoscopic gastric bypass: A case report and review of the literature

**DOI:** 10.1016/j.ijscr.2022.106819

**Published:** 2022-02-08

**Authors:** Ryan Nicholas, Sidiyq Mohammed, Nigel Bascombe, Dilip Dan

**Affiliations:** a Department of Clinical Surgical Sciences, University of the West Indies, St Augustine, Trinidad and Tobago; b North West Regional Health Authority, Port of Spain, Trinidad and Tobago

**Keywords:** LRYGB, Laparoscopic Roux-en-Y Gastric Bypass, DJ, Duodeno-Jejunal, CC, Caeco-colic, Malrotation, Twins, Discordant, Case report, Bariatric, Gastric bypass

## Abstract

**Introduction and importance:**

Intestinal Malrotation is an uncommon entity in the adult population; more so in monozygotic twins, where concordance is expected. In literature, discordant intestinal Malrotation has only been discovered when one twin became symptomatic, and the other was screened. To the best of our knowledge, this is the first documented case of discordant adult type intestinal Malrotation in otherwise asymptomatic monozygotic twins discovered incidentally during Laparoscopic Roux-en-Y Gastric bypass (LRYGB).

**Case presentation:**

Twins A and B met the NIH criteria for bariatric surgery, neither having symptoms of acute or chronic volvulus or history of intraabdominal surgery. Twin A had a LRYGB performed by a trained bariatric surgeon, noting no anatomic anomalies. 5 months later, Twin B had LRYGB and intestinal Malrotation was diagnosed incidentally.

**Clinical discussion:**

Diagnosis of Intestinal Malrotation is rare in adults, usually discovered after becoming symptomatic or during abdominal imaging for another indication. Two cases of discordant intestinal Malrotation in monozygotic twins have been documented, both discovered when one twin became symptomatic due to acute volvulus, suggesting epigenetic phenomena. When discovered incidentally during surgery, patients can safely undergo their intended procedure, but literature suggests prophylactic division of Ladd's bands, while appendectomy is left to the discretion of the surgeon.

**Conclusions:**

Intestinal Malrotation appears to be associated with epigenetic phenomena and if discovered incidentally during surgery, the proposed procedure can be carried out by an experienced surgeon, in addition to division of Ladd's bands and appendectomy.

## Highlights

• Discovering Intestinal malrotation is rare in adults.
• Concordant intestinal malrotation is expected in monozygotic twins.
• Incidental finding of discordant intestinal malrotation in monozygotic twins.
• Discovered during laparoscopic Roux-en-Y Gastric Bypass on second twin.
• We report the first documented case.

## 1 Introduction

Malrotation is a rare occurrence in the general population, with a prevalence of 0.2–1.0%, and one in twenty-five hundred live-born infants [1]. Discordant intestinal Malrotation in twins, a much rarer entity, is typically discovered when one twin presents with a surgical emergency [2], [3]. Adult presentation accounts for 0.2–0.5% of all cases, of which 15% present with midgut volvulus [4]. A literature review revealed few cases of Malrotation in twins and even fewer cases of discordant Malrotation in homozygous twins.

To the best of our knowledge, there have been two documented cases of isolated discordant Malrotation in homozygous twins, both discovered when screening the asymptomatic twin after their twin presented with midgut volvulus. The first case involved an infant, and the second involved a 33-year-old female [2], [3]. Vidal et al. reported an incidence of incidentally diagnosed Malrotation of 0.025% in a study of 20,000 patients who underwent laparoscopic Roux-en-Y gastric bypass, none of whom were twins [5]. We present the first documented case of asymptomatic homozygous twins (A and B), who underwent LRYGB and had incidental discovery of isolated intestinal Malrotation made intraoperatively on twin B.

This case report has been reported in line with the SCARE Criteria. [6]

## 2 Case presentation

A pair of 30-year-old, homozygous twins, A and B, (BMI of 41.6 and 36.7, respectively), both gave a history of obesity, with obstructive sleep apnoea and gastroesophageal reflux disease and met the NIH criteria for bariatric surgery after comprehensive multi-disciplinary team evaluation [7]. Twin A had no prior surgical history. Twin B had L5 discectomy three months before this surgery, but no history of intraabdominal surgery. Before the operation, there was no need for abdominal imaging except routine ultrasound to rule out gallstones [8]. Neither patient had symptoms of acute or chronic volvulus indicative of intestinal Malrotation.

**Fig. 1:**
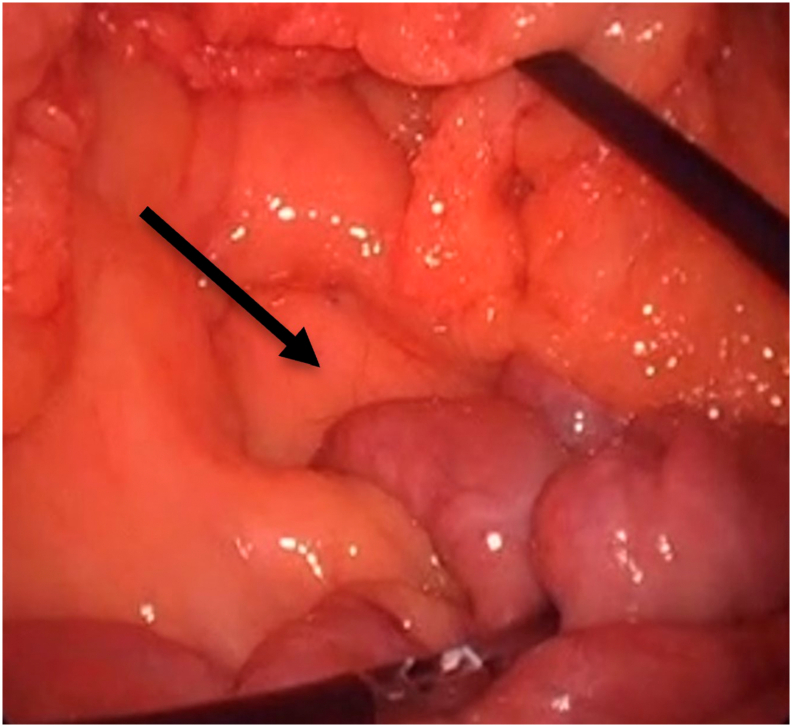
Demonstration of the duodenal-jejunal junction at the base to the transverse mesocolon in twin A.

Twin A had a standard LRYGB performed by a board-certified bariatric surgeon who noted no anatomic anomalies (Fig. 1). The procedure and the post-operative period were uneventful.

Five months later, Twin B underwent LRYGB using a standard 6-port technique by the same surgeon. The technique used creates the gastric pouch first, followed by the jejuno-jejunostomy, then the ante-colic gastro-jejunostomy.

**Fig. 2:**
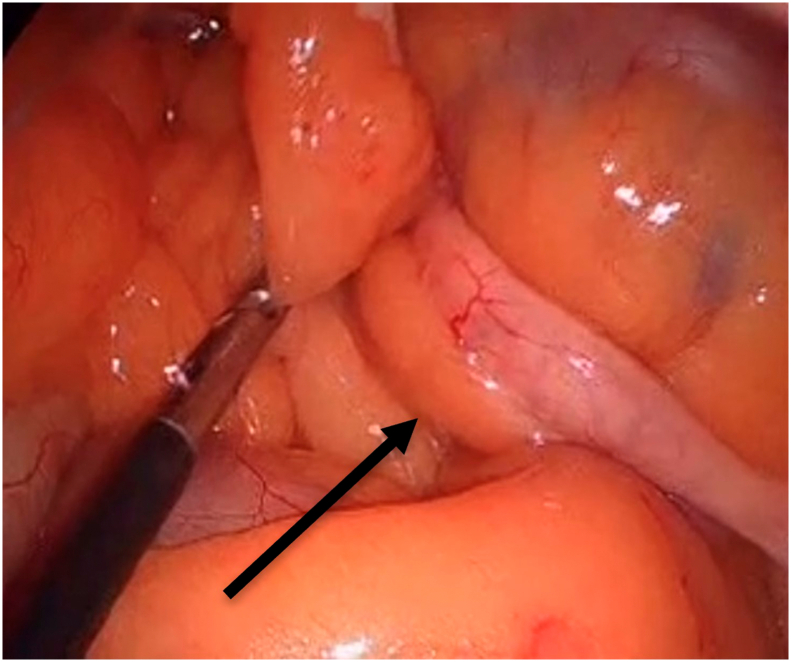
The terminal ileum with Jackson's veil and the appendix is found at the base of the transverse mesocolon in twin B.

In preparation for the Roux and Y limbs, the small bowel was examined in search of the DJ flexure. There was difficulty whereby the DJ flexure was not easily identifiable when elevating the transverse colon (Fig. 2). The appendix and cecum were in the central abdomen towards the left and the small bowel to the right. The ileum starting at the ileocecal junction was followed proximally to locate the DJ flexure to the right of the mesenteric vessels. Ladd's band was visualized and lysed, and an appendectomy was performed. Starting at the DJ flexure, 150 cm Bilio-pancreatic limb was made, and following the jejuno-jejunostomy, the Roux-en-Y gastro-jejunostomy was completed in ante-colic fashion.

Considering the intraoperative findings, the patient's history was retaken, but there were no findings suggestive of malrotation, with unremarkable family, genetic and psychosocial histories.

**Fig. 3:**
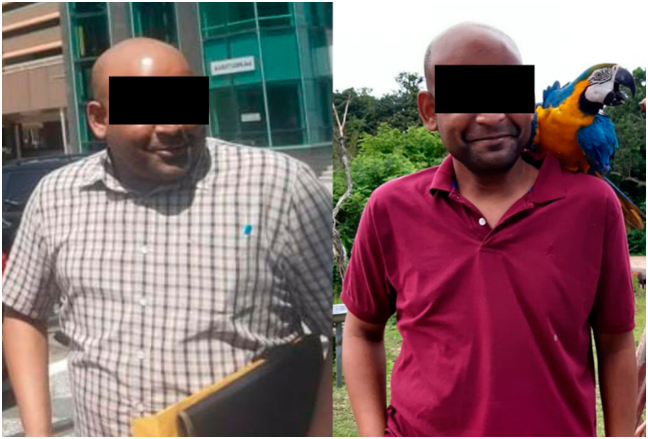
Twin A Before (Left) and after (right) operation.

**Fig. 4:**
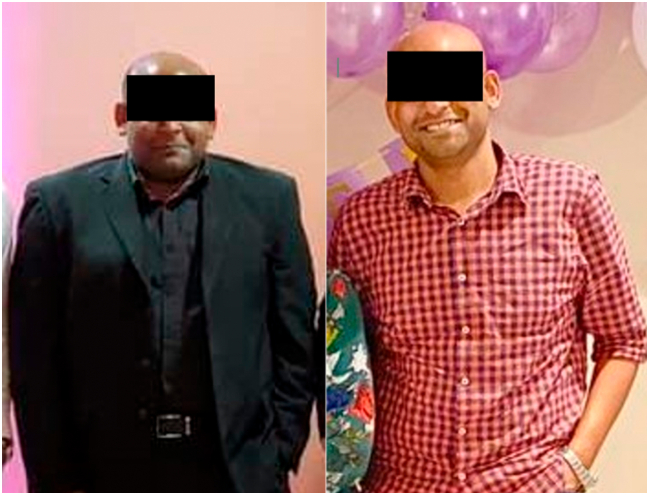
Twin B Before (Left) and after (Right) operation.

At 12 months post procedures, both patients saw the desired weight loss (Fig. 3, Fig. 4) and the reduction of the symptoms they had before surgery.

## 3 Discussion

After an extensive literature search, we present the first documented case of incidentally discovered, discordant, isolated intestinal Malrotation in asymptomatic adult twins during laparoscopic surgery. Malrotation in adults accounts for 0.2–0.5% of cases, of which 15% present with midgut volvulus [4]. A UK review by Aboagye et al. [9] found that the peak incidence of intestinal malrotation was in the first month of life (30%), with 75% of cases presenting by age five. Adult presentations can be incidental (asymptomatic but undergo imaging/laparotomies for alternative indications), chronic (insidious with nonspecific symptoms such as cramping abdominal pain, bloating, weight loss) and acute (acute midgut volvulus) [4]. The true prevalence of asymptomatic Malrotation is unknown.

Under normal circumstances, the bowel rotates around the superior mesenteric artery in stages. Stage one (5–10 weeks gestation) involves physiological herniation of the bowel and anticlockwise rotation of the DJ loop and the CC loop 180^o^ and 90^o^, respectively. In stage two, the bowel returns into the abdominal cavity (week 10). The Duodeno-Jejunal and Caeco-colic loops continue to rotate, both completing 270^o^ total rotation. In Stage three (week 11 onward), the cecum descends to the right lower quadrant, and there is fixation of the mesenteries. Abnormalities of rotation are classified as nonrotation (Arrest in Stage One), incomplete rotation (Arrest in stage Two, which can lead to duodenal obstruction and involves Ladd bands forming from the misplaced cecum across the duodenum), or incomplete fixation (Arrest in Stage Three). All carry the risk of midgut volvulus [10].

The aetiology of intestinal Malrotation is incompletely understood, but authors have suggested a genetic component. A review by Martin et al. suggested the involvement of heterozygous mutations in the forkhead transcription factor FOXF1 and mutations in genes controlling L-R patterning; however, these are associated with other specific syndromic changes [11]. They also cited several case reports of autosomal dominant inheritance patterns of non-syndromic (isolated) intestinal Malrotation, but no single genetic abnormality has yet been identified [11]. The syndromic associations with Malrotation are beyond the scope of this paper.

Intestinal Malrotation in monozygotic twins is rare. Kikuchi et al. first documented a case of concordant Malrotation in 1977, in new-born twins presenting with bilious vomiting [12]. Crowley et al. report a case of isolated concordant Malrotation in twin neonates, one of whom again presented with acute volvulus [13]. Other cases found had syndromic associations or other congenital abnormalities.

Two cases of discordant intestinal Malrotation in monozygotic twins have been reported. Smith et al. (2005) [2] report a case of a neonate presenting at one week of age with midgut volvulus; a Ladd procedure was performed, with no other abnormalities noted. Screening of the second twin via upper GI contrast study revealed normal rotation. Bourgouin et al. (2015) [3] reports a 33-year-old female presenting with midgut volvulus, with a history of chronic abdominal pain from childhood and nil prior surgeries. A Ladd's procedure was performed. The second twin underwent a screening CT revealing normal rotation. These findings suggest that epigenetic phenomena may be responsible for intestinal Malrotation, in addition to the autosomal dominant and recessive patterns previously described.

Levin et al. suggest screening of the asymptomatic twin when Malrotation is discovered in the other. The potential loss of bowel offsets the additional cost, and potential risk of radiation from screening should a midgut volvulus occur [14]. Graziano et al. recommend an upper GI contrast study for diagnosing Malrotation in a pediatric population [15]. In adults, the investigation of choice is contrast-enhanced CT [16].

Typically, patients diagnosed during adulthood are asymptomatic or have a history of chronic abdominal pain and vomiting (likely due to chronic partial obstruction). [4] Few instances of Malrotation discovered during bariatric surgery have been documented, and less than 100 cases of adult malrotation. Routine abdominal imaging is rarely performed before gastric bypass surgery, and routine upper GI series can be omitted from the preop evaluation for bariatric surgery [8]. Several surgeons, instead of examining the Duodeno-jejunal flexure first, prefer to start with the construction of the gastric pouch, then move to the intestinal part of the procedure, which can produce an unpleasant surprise should Malrotation then be discovered [5].

Controversy exists concerning the proper management of asymptomatic patients in whom Malrotation is discovered incidentally during imaging/surgery for another indication, as the risks of corrective procedures in this population are not known [17]. These patients can safely undergo bariatric surgery laparoscopically, with few instances of procedure modification being required [5], [18]. Although volvulus due to malrotation does occur in adults, it is a rare event, and if discovered via imaging, most patients should be carefully observed rather than have a Ladd procedure performed, particularly if discovered after the age of 20 [19]. However, when discovered intraoperatively, including during laparotomies and laparoscopic bariatric surgery, such as LRYGB, evidence suggests prophylactic division of Ladd's bands. Due to recent advances in cross-sectional imaging, concurrent appendectomy is not required; however, this is left up to the surgeon's discretion, who may perform appendectomy due to the atypical location of pain should appendicitis occur later. We believe appendectomy should be performed to avoid confusion in gastric bypass patients with malrotation. Laparoscopic sleeve gastrectomy is a reasonable alternative to LRYGB in these circumstances due to the potential difficulty of creating a tension free gastrojejunal anastomosis [20].

Our patient was asymptomatic and 30 years old, and we opted to perform LRYGB, division of Ladd bands and an appendectomy.

## 4 Conclusion

Malrotation, though once thought to result from specific genetic abnormalities and autosomal inheritance patterns, appears to be associated with epigenetic phenomena. Evidence supports screening an asymptomatic twin if Malrotation is discovered in the other. If asymptomatic malrotation is discovered before the age of 20, a prophylactic Ladd's Procedure is warranted. When this is discovered during bariatric surgery, performing the proposed procedure is generally safe but should be performed by an experienced surgeon, should difficulty be encountered. Ladd Bands should be divided where possible, and an appendectomy may be performed at the surgeon's discretion.

## Sources of funding

None.

## Ethical approval

This report is not a research study.

## Consent

Written informed consent was obtained from the patient for publication of this case report and accompanying images. A copy of the written consent is available for review by the Editor-in-Chief of this journal on request.

## Guarantor

Dr. Ryan Nicholas.

## Provenance and peer review

Not commissioned, externally peer-reviewed.

## CRediT authorship contribution statement

Dr Ryan Nicholas: Conceptualization, Writing- original draft, review and editing

Dr Sidiyq Mohammed: Writing- Review and editing

Dr Nigel Bascombe: Visualization

Dr Dilip Dan: Surgeon who performed operations and follow-up, Supervision, Project Administration, Writing- Review and Editing

## Declaration of competing interest

The authors have no conflicts of interest to declare.
